# The current situation of *Angiostrongylus vasorum* in Romania: a national questionnaire-based survey

**DOI:** 10.1186/s12917-021-03034-1

**Published:** 2021-10-07

**Authors:** Georgiana Deak, Eduardo Berriatua, Andrei Daniel Mihalca

**Affiliations:** 1 Department of Parasitology and Parasitic Diseases, Faculty of Veterinary Medicine, University of Agricultural Sciences and Veterinary Medicine of Cluj-Napoca, Calea Mănăștur 3-5, 400372 Cluj-Napoca, Romania; 2 Department of Animal Health, Faculty of Veterinary Science, Regional Campus of International Excellence ‘Campus Mare Nostrum’, University of Murcia, 30100 Murcia, Spain

**Keywords:** Awareness, *Angiostrongylus vasorum*, Distribution, Lungworms, Questionnaire, Romania

## Abstract

**Background:**

*Angiostrongylus vasorum* (Nematoda, Metastrongyloidea) is a vascular nematode that resides in the pulmonary arteries and the right side of the heart of a wide variety of carnivores, with an indirect life cycle using coprophagic gastropods as intermediate hosts. For domestic dogs, the infection with *A. vasorum* can be asymptomatic, but more frequently, it is associated with a wide range of clinical manifestations like cardio-respiratory signs, bleedings, neurological signs, and ocular problems which can lead to death when not treated accordingly. Angiostrongylosis was confirmed for the first time in Romania in red foxes (*Vulpes vulpes*) in 2017 and two years later a seroepidemiologic study was conducted among domestic dogs. However, to this date, no clinical canine angiostrongylosis cases were published in Romania. The aim of the present paper was to evaluate the knowledge about canine angiostrongylosis among veterinarians in Romania and to update the distribution of this disease using a national wide anonymous questionnaire.

**Results:**

Overall, 147 unique responses were submitted, from 31 out of 42 counties. Twelve veterinarians (8%) from 8 counties (26%) acknowledged diagnosing a case of angiostrongylosis including 5 from the Bucharest and 1 from each of the remaining seven counties. All affected dogs had respiratory distress, 75% suffered cardiopathy, 16% coagulopathies and 8% neurological signs. Case diagnosis was based mostly on larval detection by coprology (67%) and serological antigen detection test (42%).

**Conclusions:**

Romanian veterinarians are aware of canine angiostrongylosis and a significant number have clinical experience with the disease. Epidemiological studies are now needed to assess its distribution in the country, and further efforts are required to improve understanding of the disease, its diagnostic and treatment methods among veterinarians.

**Supplementary Information:**

The online version contains supplementary material available at 10.1186/s12917-021-03034-1.

## Background

*Angiostrongylus vasorum* (Nematoda, Metastrongyloidea) is a vascular nematode that resides in the pulmonary arteries and the right side of the heart of a wide variety of carnivores, mostly canids (foxes, jackals, wolves, domestic dogs) and occasionally in other animal species [1–3]. It has an indirect life cycle using coprophagic gastropods (snails and slugs) as intermediate hosts. Adult female nematodes are ovoviviparous, they lay eggs that hatch quickly, and the first-stage larvae (L1) circulate through the pulmonary capillaries, penetrate the alveoli, and then migrate to the oropharynx where they are swallowed and released into the environment via faeces [2–4]. Carnivores get infected after the accidental ingestion of the third-stage larvae together with the intermediate hosts, their slime, or paratenic hosts. In the definitive hosts, L3 migrate via blood/lymph vessels to the pulmonary arteries or right heart where they become adults, after two successive molts in the mesenteric lymph nodes [5–8]. For domestic dogs, the infection with *A. vasorum* can be asymptomatic, but more frequently, it is associated with a wide range of clinical signs. Among these, the most common ones are the cardio-respiratory manifestations, bleedings, neurological signs, and ocular problems which can lead to death when not treated accordingly [9–13]. *Angiostrongylus vasorum* (Baillet 1866) was first described in France, but nowadays it is emerging in Europe [13–15] In 2009, Morgan [16] elaborated a climatic model for *A. vasorum* which included western Romania as a potential area for the presence of angiostrongylosis. Almost ten years later, *A. vasorum* was confirmed for the first time in Romania in red foxes (*Vulpes vulpes*) with 4.2% prevalence [17]. Two years later, a second study was conducted in order to evaluate the seroepidemiologic status of *A. vasorum* among domestic dogs with a seroprevalence of 2.14% for antigens or antibodies [18]. However, to this date, no clinical canine angiostrongylosis cases were published in Romania.

With this in view, our aim was to evaluate the level of understanding and clinical experience of veterinarians from Romania about canine angiostrongylosis. This is considered important to assess the need of promoting awareness on the disease and to perform further epidemiological studies to analyze its distribution in Romania.

## Results

Overall, 147 unique responses were submitted (29.4%) out of 500 targeted, from 31 (73.8%) out of 42 counties. The highest frequency of the answers was from Cluj County (18.36%), followed by Bucharest (16.32%), Iași (7.48%) and Timiș (4.76%), all large cities with veterinary medicine faculties. Study results are available in the supplementary material 2. The participation percentage among veterinarians on social groups was 3.56, 3.8 and 3.41% respectively and represented 1.36% of the total number of registered veterinarians in Romania (*n* = 10,814) (www.cmvr.ro).

**Fig. 1:**
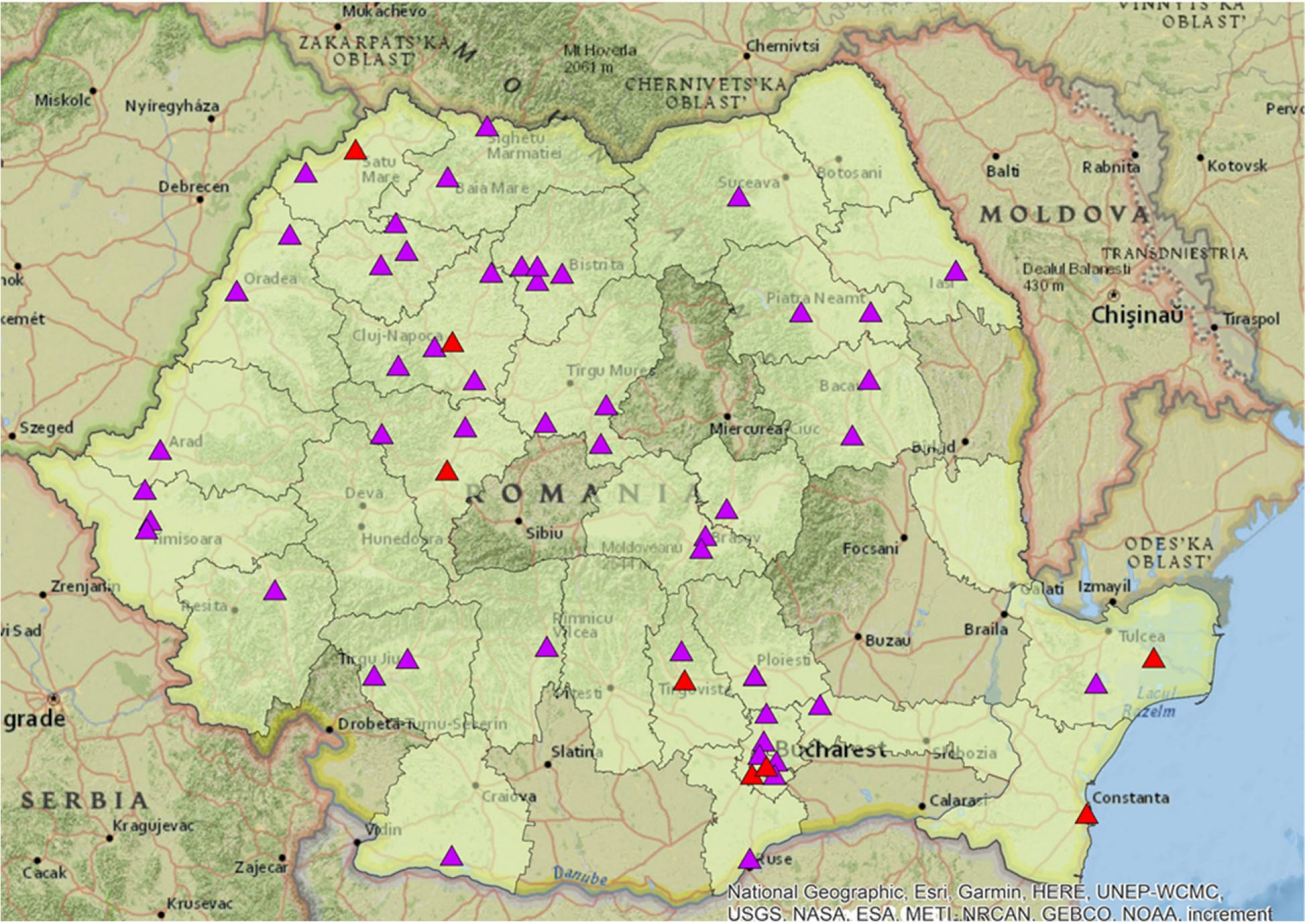
The representation of counties from which responses were recorded and the distribution of positive responses. Purple triangles: veterinarians that heard about angiostrongylosis; Red triangles: *A. vasorum* positive cases

The proportion of veterinarians familiar with angiostrongylosis was 83.7% (*n* = 123) and varied between regions (Fig. 1). Most of them learned about this disease during their academic studies and from pharmaceutical companies (77.2% each) (*n* = 95), followed by participation in conferences (37.4%) (*n* = 46), continuous training courses (35.8%) (*n* = 44), scientific articles (35.8%) (n = 44) and from other colleagues (23.6%) (*n* = 29). Among the veterinarians that were aware of canine angiostrongylosis, 68.9% (*n* = 84) would consider angiostrongylosis as a potential diagnosis in a patient presenting cardio-respiratory, neurological or coagulopathy signs. Out of these 84, 47.6% (*n* = 40) would do the Baermann larval migration diagnostic method to confirm the diagnosis, 31% (*n* = 26) would send a sample to a specialized diagnostic lab, 23.8% (*n* = 20) would use diagnostic imagery and 20.2% (*n* = 17) would carry out an antigen detection test, and 16.7% (*n* = 14) would not do any test to confirm their clinical suspicions.

Twelve veterinarians (8.5%) from eight counties admitted having diagnosed a case of canine angiostrongylosis in Romania and 5 came from Bucharest compared to 1 from each of the remaining seven counties. Clinical signs reported in order of frequency were respiratory distress (100%), cardiological conditions (75%), coagulopathy (16.7%) and neurological (8.3%), and 16.7% reported all four clinical signs. The frequency of diagnostic tests used included 66.7% the Baermann larval migration method, 41.7% the antigen detection AngioDetect (IDEXX) rapid test, 25.0% post-mortem examination, 16.7% imagery and 8.7% molecular tests. Among 8 treated dogs, 7 (85.7%) received macrocyclic lactones and 1 dog (12.5%) was treated with melarsomine. The complete statistical analysis is available in the supplementary material 3.

## Discussion

The results of the present survey provide important information on the knowledge of canine angiostrongylosis among veterinarians in Romania, where this parasite was first described in red foxes only 4 years ago [17] when it was detected in 4 counties out of 10 checked. However, in the present case, angiostrongylosis was not diagnosed in Mureș, Hunedoara and Sălaj counties, areas in which angiostrongylosis was confirmed in foxes [17]. In a later serological study, seroprevalence was highest in Hunedoara (6.06, 95% CI - 2.94–10.86) and Harghita counties (5.77, 95% CI - 1.21–15.95) [18], but in the present survey we had no responses from Harghita county and no reported cases in Hunedoara County. Questionnaire participation was relatively small and some reporting bias cannot be excluded. Veterinarians familiar with the disease were possibly more likely to answer the questionnaire compared to other veterinarians. Consequently, our results may not fully represent the country’s veterinary communities understanding of Angiostrongylosis. The disease has a patchy spatial distribution [13, 19–21]and this also seems to apply to Romania [13, 19–21]. However, further clinical and epidemiological studies need to be carried out to provide a more accurate picture of the current distribution and infection rate in the population of Romanian dogs.

Even though disease surveillance using questionnaires has limitations, they may be used for investigation of emerging pathogens and for raising awareness among veterinarians as aimed with the present paper [22]. The lack of interest in participating in these studies may be related to the lack of knowledge on this topic or to the lack of interest. As the questionnaire was especially conceived to take maximum 2 min to answer it, the lack of time can be excluded as a motive for the low response rate. However, even though we did not have as many answers as aimed, the responses are very important for offering an overview and update the distribution of angiostrongylosis in our country. As shown in Fig. 1, most of the vets that responded are from large cities, around universities, and we hypothesize that they may manifest a bigger interest in being updated. Our results proved that veterinarians from Bucharest tend to be more informed. This may be related to the high-rate participation of vets from the capital to various training courses in this field. Also, we sustain the previously reported idea that veterinarians that have seen or had cases of angiostrongylosis, were more likely to respond to this questionnaire [23]. Unfortunately, we obtained less than 5 answers from rural areas, possibly indicating a limited interest of this category of vets in parasitic diseases of pets. Compared to other parasitological-related questionnaire studies made in Romania that also targeted veterinarians (122 answers) [24], we had a higher number of responses (147) from the same number of counties.

Angiostrongylosis represents a severe emerging disease of canids in Europe, and there is insufficient information about it in Romania. Auspiciously, the present data highlights the importance of updating the academic curricula to include emerging diseases but also the importance of pharmaceutical companies in veterinary education as a by-product of commercial promotions. Unfortunately, due to the lack of awareness, previous studies from Romania tend to neglect infection with lungworms in dogs by not performing larval migration tests necessary to detect these infections [25]. Similarly, private practitioners tend not to use the Baermann method in dogs (Mihalca - personal observation). Infection with *A. vasorum* should be suspected based on the clinical signs, but confirmation of the diagnosis requires the identification of the first stage larvae (L1) in the respiratory tract or in the faeces of the infected dog, using broncho-alveolar lavage, or Baermann migration technique which is the gold standard method [2]. Notwithstanding this, the sensitivity of these methods is limited because clinical signs may appear during the prepatent period and larval shedding during patency is intermittent [26]. Notably, a negative fecal examination was reported in a dog with ocular angiostrongylosis [11]. Diagnostic sensitivity can be increased if the samples are collected from three consecutive days [10]. The detection of L1 larva via bronchoalveolar lavage it is not widely used, and it’s usually unsuitable for dyspneic patients [27]. The Angio Detect rapid blood test (IDEXX Laboratories, Westbrook, ME, USA) for antigen detection has become popular because it is easy to use and not expensive and claims a sensitivity of 84.6% [28] and a specificity of 100%. The possibility of false negative results using Angio Detect should encourage veterinarians to use complementary techniques for diagnosing canine angiostrongylosis, especially in symptomatic patients [27]. A quantitative PCR test can be used on different samples, but usually it has a low sensitivity [19, 29, 30] b. Histological examination can reveal the presence of coiled larvae and adult nematodes in the lung’s parenchyma and pulmonary arteries for the post-mortem diagnosis [31, 32]. Diagnostic imagery is a valid diagnostic option and one of the most recent studies in this field showed a high sensitivity (100%) and specificity (92%) of thoracic ultrasound in detection of angiostrongylosis in dogs with pulmonary distress [8].

Macrocyclic lactones are highly efficacious treatments for canine angiostrongylosis and was the treatment choice of all except one (out of 8) of the veterinarians that answered this questionnaire who reported using melarsomine, a treatment against adult cardiac *Dirofilaria immitis* nematodes. Unfortunately, in regard with the present study and our personal discussions with veterinarians from Romania, often reflect confusion between *D. immitis* and *A. vasorum*, especially when diagnosis is made using echocardiography. This highlights the importance of promoting awareness of this disease in the population of Romanian canids among veterinarians and dog owners and to support continuous training programs. With the present paper we are hoping to contribute in this respect.

## Conclusions

A large proportion of Romanian veterinarians who responded to the questionnaire are aware of canine angiostrongylosis and a significant number have clinical experience with the disease. Epidemiological studies are now needed to assess its distribution in the country, and further efforts are required to improve understanding of the disease and diagnostic and treatment methods among veterinarians.

## Methods

A short anonymous questionnaire was developed using the tool provided by www.jotform.com and distributed to veterinary clinicians, private and rural practices, using social media platforms (Instagram, Facebook) and e-mail addresses, between 12 of February and 12 of March 2021 [33]. We aimed to get around 500 answers in 4 weeks. On Facebook, we used three closed groups for veterinarians. One had 4123 members, the second 3868 members and the last 4307 members. The questionnaire was sent individually on the email addresses available on the official website www.cmvr.ro. The invitation message referred to veterinarians practicing in Romania. Weekly reminders were sent. Before releasing the questionnaire online, it was tested by 4 independent veterinary researchers from our team to clarify any eventual errors or reformulate unclear questions. The questions were in Romanian language. First, the aim of the study was shortly described together with a request for asking participants to answer based on their current knowledge only, without using external information resources. The questionnaire included a section on general information regarding the veterinarians’ location followed by 8 close-ended qualitative (yes or no answers) questions addressing their training and understanding of the clinical signs associated with angiostrongylosis and their clinical experience, diagnostic and treatment choices and outcome of the disease (Additional file 1).

The data was incorporated in a Microsoft Excel sheet and the proportion, standard error and 95% confidence interval of question responses were calculated. Fisher’s exact test was used to compare proportions taking for significance *p* < 0.05 for a two-sided test. The distribution map based on the questionnaire answers was generated using ArcMap 10.6.1.

## Supplementary Information

**Additional file 1.**

**Additional file 2.**

**Additional file 3.**

## Data Availability

All data generated or analyzed during this study are included in this published article and its supplementary information files.
