# Supplementary material for: The current situation of Angiostrongylus vasorum in Romania: a national questionnaire-based survey
Source: BMC Vet Res. 2021 Oct 7;17:323. doi: 10.1186/s12917-021-03034-1 (PMC8496003; doi:10.1186/s12917-021-03034-1)
Supplement: Supplementary file 1 — Additional file 1. [file 12917_2021_3034_MOESM1_ESM.docx]

Questionnaire for veterinary doctors

The present anonymous questionnaire refers strictly to veterinarians and aims to evaluate the knowledge about canine angiostrongylosis. If you chose to answer this it, please do not use any external information sources to answer.

1. Did you ever hear about canine angiostrongylosis produced by *Angiostrongylus vasorum*?
   1. Yes
   2. No
2. Where did you hear about it?
   1. Faculty
   2. Continuous training
   3. Conferences/Symposiums/Congresses/Workshops
   4. Pharmaceutical companies
   5. Discussions with other veterinarians
   6. I don’t know/I don’t want to answer
   7. Other places
3. Would you consider angiostrongylosis as a potential diagnosis if you have a patient with respiratory/cardio-vascular/neurological/bleeding symptoms?
   1. Yes, and I do larvoscopy
   2. Yes, and I test it with AngioDetect rapid test
   3. Yes, and I send a sample to a specialized lab
   4. Yes, but I don’t do anything to confirm it
   5. Yes, and I ask for medical imaging
   6. No
   7. I don’t know/I don’t answer
4. Did you diagnose canine angiostrongylosis?
   1. Yes
   2. No
5. Which were the main symptoms?
   1. Cardio-vascular
   2. Respiratory
   3. Neurological
   4. Coagulopathies
   5. Other
6. What method did you use to confirm the diagnostic?
   1. Larvoscopy
   2. Serology (AngioDetect)
   3. Molecular biology
   4. Necropsy
   5. Other
7. What therapy did you administer?
8. Treatment outcome
   1. The patient cured
   2. The patient died
   3. Other situations

By submitting this form, you agree and consent to your personal data which you provide to the USAMV Cluj-Napoca to be processed and used.
